# Post-marketing safety profile of meningococcal group B vaccines: a real-world disproportionality analysis of the VAERS database, 2015Q1–2025Q3

**DOI:** 10.3389/fphar.2026.1745876

**Published:** 2026-03-18

**Authors:** Guojun Liang, Hao Huang, Jihui Huang, Qiong Liu, Yang Song

**Affiliations:** Huadu District People’s Hospital of Guangzhou, Guangzhou, Guangdong, China

**Keywords:** meningococcal vaccines, *Neisseria* meningitidis serogroup B, pharmacovigilance, vaccine safety, VAERS

## Abstract

**Background:**

Serogroup B meningococcal (MenB) disease remains a serious public health concern. Recombinant protein-based vaccines have been licensed since the mid-2010s, but ongoing surveillance is needed to capture rare or long-term adverse events.

**Methods:**

We analyzed reports submitted to the Vaccine Adverse Event Reporting System (VAERS) from 2015Q1 to 2025Q3,with data available through 30 September 2025. Cases explicitly identifying MenB vaccines were included. Events were coded with MedDRA, and disproportionality analyses were conducted using Reporting Odds Ratio (ROR), Proportional Reporting Ratio (PRR), and Empirical Bayes Geometric Mean (EBGM). Subgroup analyses assessed differences by age, sex, seriousness, and coadministration.

**Results:**

A total of 16,611 reports were identified, of which 19.9% were serious. Most events reflected expected reactogenicity such as fever, syncope, headache, and injection site reactions. Several unlabeled events, including pallor and hypotonia, showed elevated RORs. Time-to-onset analyses demonstrated clustering within 2 days of vaccination across all strata, with serious events occurring earlier than non-serious events. No novel syndromic clusters were observed.

**Conclusion:**

MenB vaccines show a favorable safety profile in real-world use. While most events align with established product information, a small number of disproportionate but unlabeled events merit continued pharmacovigilance.

## 1 Introduction

*Neisseria* meningitidis serogroup B is a leading cause of invasive meningococcal disease worldwide, particularly among infants, adolescents, and young adults. Although the global incidence of meningococcal disease has declined over recent decades due to widespread vaccination against serogroups A, C, W, and Y, serogroup B continues to contribute substantially to morbidity and mortality, especially in high-income countries where it accounts for many cases among younger age groups (Shen et al., 2024; Olbrich et al., 2018). The disease’s unpredictable epidemiology, rapid progression to meningitis or septicemia, and potential for severe complications such as neurological sequelae and death underscore its public health significance (Marshall et al., 2024).

The development of effective vaccines against serogroup B posed substantial challenges. Its capsular polysaccharide is poorly immunogenic and structurally similar to human neural cell adhesion molecules, raising concerns about autoimmunity and limiting the feasibility of traditional polysaccharide-based vaccines (Bartoloni et al., 1995). To overcome these obstacles, protein-based recombinant vaccines were developed to include conserved surface antigens that can elicit broad protective responses (Howitz et al., 2007; Ruiz et al., 2021). Bexsero, first developed by Novartis and later produced by GlaxoSmithKline, contains multiple antigens including factor H binding protein, *Neisseria* adhesin A, PorA P1.4 outer membrane protein, and neisserial heparin binding antigen, designed to protect against a broad spectrum of circulating strains (Deghmane and Taha, 2022). Trumenba, developed by Pfizer, employs a bivalent formulation targeting two distinct factor H binding protein variants to broaden strain coverage. Both vaccines were licensed in North America, Europe, and other regions beginning in the mid-2010s, marking a pivotal milestone in the prevention of serogroup B meningococcal disease.

Despite these advances, pre-licensure clinical trials have inherent limitations in evaluating vaccine safety. Such trials are conducted in controlled settings and typically enroll selected populations, with sample sizes that are sufficient to identify common reactions but too limited to detect rare clinical outcomes. Follow-up periods are generally short, which restricts the detection of delayed effects, and the enrolled participants may not represent the full spectrum of real-world recipients, such as those with chronic illnesses or concomitant medication use. Therefore, post-licensure surveillance is indispensable for ensuring comprehensive safety evaluation (Moro et al., 2019; Griffin et al., 2009).

In the United States, the Vaccine Adverse Event Reporting System (VAERS) serves as a cornerstone of vaccine safety monitoring. Jointly managed by the Centers for Disease Control and Prevention and the Food and Drug Administration, VAERS collects spontaneous reports of adverse events following vaccination from healthcare providers, manufacturers, and the public. Its principal strength lies in its capacity to detect potential safety signals rapidly within a large and heterogeneous population (Shimabukuro et al., 2015). At the same time, VAERS has limited sensitivity for identifying very rare or clinically complex serious outcomes, particularly when reporting is incomplete or delayed, a limitation that is especially relevant for analyses seeking to characterize uncommon severe events. However, as a passive reporting system, VAERS is constrained by several inherent limitations, including underreporting, variability in data quality, susceptibility to reporting bias, and the absence of reliable denominators for incidence estimation. Consequently, VAERS data cannot establish causal relationships and are primarily used to generate hypotheses for further evaluation through active surveillance systems or controlled epidemiologic studies.

Several post-licensure evaluations of MenB vaccine safety have already been conducted using VAERS data. Duffy et al. analyzed reports following the MenB-FHbp vaccine from licensure in October 2014 through December 2018, characterizing recipient age and sex distributions, coadministration with other vaccines, and adverse events coded using MedDRA; they found that the most commonly reported events were expected reactogenicity (e.g., fever, headache, injection-site pain), serious events were rare, and no new safety signals were identified (Duffy et al., 2020). Similarly, Perez-Vilar et al. reviewed VAERS reports for MenB-4C from January 2015 through December 2018, observing frequent local and systemic reactions, very few serious reports, and alignment with known vaccine safety profiles. Both studies used descriptive analytics and standard signal detection/data-mining approaches appropriate to passive surveillance, concluding that early post-licensure safety data were consistent with expected reporting patterns (Perez-Vilar et al., 2022). However, their findings are constrained by limitations: the reporting period ends in 2018, spontaneous reporting may under-capture rare or delayed events, data completeness is variable, and they are unable to assess long-term trends, evolving patterns of vaccine use, or emergence of very rare adverse outcomes.

The present study aims to address the limitations of previous post-licensure evaluations by providing a more comprehensive assessment of MenB vaccine safety in the United States. Building on earlier work that was restricted to data collected before 2018, this analysis draws on a substantially larger and more recent dataset encompassing both licensed products to enable broader characterization of reported adverse events. It focuses on describing demographic and clinical patterns of reported cases, characterizing product-specific reporting patterns, and on identifying potential safety signals through systematic evaluation of reporting trends over an extended observation period spanning both the pre-pandemic and pandemic eras. In addition, particular attention is given to the detection and clinical interpretation of potentially unlabeled adverse event signals identified through systematic disproportionality analyses. Given the intrinsic constraints of passive surveillance, the objectives of this study are explicitly hypothesis-generating, intended to identify patterns that warrant further investigation rather than to establish causality. By adopting a longer follow-up horizon and expanding the analytic scope beyond prior descriptive studies, this study seeks to provide a clearer understanding of the real-world safety profile of MenB vaccines.

## 2 Methods

### 2.1 Data source and case selection

This study was based on reports submitted to the Vaccine Adverse Event Reporting System (VAERS), a national passive surveillance database jointly managed by the Centers for Disease Control and Prevention (CDC) and the Food and Drug Administration (FDA). The database collects reports of adverse events following immunization from healthcare providers, manufacturers, and members of the public. Reports from the first quarter of 2015 through the third quarter of 2025 (with data available through 30 September 2025) were obtained from the official VAERS website (https://vaers.hhs.gov) and processed according to the procedures described in the VAERS Data Use Guide published by CDC/FDA.

Only domestic reports that explicitly specified the administered vaccine brand as either Bexsero (MenB-4C) or Trumenba (MenB-FHbp) were included. Reports were excluded if they contained no coded adverse event terms, originated outside the United States, were identified as duplicates based on matching VAERS ID and vaccination date, or lacked vaccine brand information. For the purpose of our disproportionality analysis, only reports containing at least one valid coded adverse event term were retained to ensure methodological consistency (Supplementary Figure S1).

### 2.2 MedDRA coding and processing

All adverse events were coded using version 27.0 of the Medical Dictionary for Regulatory Activities (MedDRA). Preferred Terms (PTs) were mapped to their corresponding System Organ Classes (SOCs) according to the standardized MedDRA hierarchy to allow structured aggregation and analysis. To ensure consistency and reproducibility, no manual modification or recoding of terms was performed, and analyses were conducted at the PT level and higher within the MedDRA structure to ensure comparability with other pharmacovigilance studies.

### 2.3 Variable definitions

Demographic variables included age and sex as recorded in the structured VAERS dataset. Sex was categorized as female, male, or unknown. Age was divided into six clinically meaningful, mutually exclusive groups reflecting developmental and immunologic stages relevant to MenB vaccination: <2 years, 2 ≤ age <3 years, 3 ≤ age <6 years, 6 ≤ age <12 years, 12 ≤ age <18 years, and ≥18 years. The first three groups represented early childhood, when immune maturation is ongoing and adverse events such as febrile seizures are more likely to occur. The 6–12-year group reflected the school-age period when MenB vaccination is less common but catch-up doses may be administered, whereas the 12–18-year group corresponded to the routine adolescent vaccination schedule. The adult group included individuals at increased risk, such as college students and military recruits. Reports with missing or implausible age values were excluded from age-stratified analyses but retained in the overall dataset.

The post-vaccination onset interval was defined as the number of days between vaccination and the first reported symptom. Reports with missing dates or negative onset intervals were excluded from time-to-onset analyses, whereas same-day onset events (interval = 0 days) were retained, but remained in disproportionality analyses.

Serious adverse events were defined according to FDA criteria: death, life-threatening illness, hospitalization or prolonged hospitalization, permanent disability, or congenital anomaly. Adverse events were also grouped by clinical system based on MedDRA SOCs to facilitate evaluation of neurological, immune, hematologic, cardiac, respiratory, gastrointestinal, dermatologic, musculoskeletal, and general disorders.

### 2.4 Clinical assessment of detected signals

All PTs meeting statistical thresholds for disproportionality were subjected to structured clinical review. Each identified PT was compared with the adverse reactions listed in the *Food and Drug Administration (FDA)* and *European Medicines Agency (EMA)*-approved product information for Bexsero and Trumenba. Events documented in either label were classified as labeled and interpreted as expected based on the established safety profiles of these vaccines. PTs not included in either label were considered unlabeled and were descriptively flagged for further clinical review, particularly when they were associated with statistically elevated reporting odds ratios or occurred in clinically relevant subgroups.

The clinical significance of detected signals was further assessed using the International Council for Harmonisation (ICH) E2A guidance on serious and medically important adverse events. This framework allowed classification of signals into categories such as immune-mediated, neurological, or hematologic disorders to support assessment of biological plausibility. Signals that overlapped categories were resolved by prioritizing those with the highest reporting frequency and clearest clinical attribution to vaccination.

### 2.5 Statistical analysis

The primary disproportionality measure was the Reporting Odds Ratio (ROR). For each vaccine–event pair, a 2 × 2 contingency table was constructed with counts of (1) the event of interest after MenB vaccination, (2) all other events after MenB vaccination, (3) the event of interest after non-MenB vaccines, and (4) all other events after non-MenB vaccines within the same study window. The non-MenB comparator pool comprised all other vaccines reported to VAERS during the same period and thus included vaccines administered across different age groups and indications. The comparison framework was designed for signal detection within a spontaneous reporting context rather than for estimation of absolute or relative risk.

This broad comparator was selected to maximize the generalizability of the analysis across all age groups for which MenB vaccines are administered and to avoid selection bias associated with choosing a specific vaccine class, while recognizing that it introduces heterogeneity into the background reporting rate and may reflect differences in age distribution, clinical indication, and reporting behavior across vaccine types.

The ROR and its 95% confidence interval were calculated on the log scale. When any cell count was zero, the Haldane–Anscombe correction was applied by adding 0.5 to all four cells. A signal was considered present when the lower bound of the 95% CI exceeded 1, consistent with WHO–UMC recommendations for signal detection (Candore et al., 2015). As a sensitivity analysis to account for multiple testing across PT-level screening, p values derived from ROR estimates were further adjusted using the Benjamini–Hochberg false discovery rate procedure, as prespecified for secondary robustness assessment.

The Proportional Reporting Ratio (PRR) was used as a supportive method. PRR signals were considered positive when PRR ≥2, chi-square >4, and the event count ≥3, in line with Evans SJW criteria (Evans et al., 2001). The Empirical Bayes Geometric Mean (EBGM) from a shrinkage model was also used as supportive evidence; an EBGM05 > 2 was regarded as supportive but not definitive evidence of a signal. In addition, a Bayesian Confidence Propagation Neural Network (BCPNN) analysis was conducted, and the Information Component lower bound (IC_025 > 0) was taken as supportive evidence (Bate and Evans, 2009; Hauben and Zhou, 2003; Zorych et al., 2013).

Subgroup analyses were prespecified to assess robustness and clinical relevance. ROR and supportive analyses were stratified by sex, by age group using the six-level schema, and by coadministration status (MenB alone vs. MenB with at least one other vaccine). Age stratification was further used to evaluate and mitigate potential confounding arising from heterogeneity in the non-MenB comparator population by enabling comparisons within more homogeneous age-defined strata. These stratified analyses were intended to partially address, but not eliminate, residual confounding inherent to the use of a heterogeneous comparator pool in passive surveillance data.

Coadministration status was inferred from the VAERSVAX file by aggregating all vaccines recorded under the same VAERS_ID, and reports listing more than one vaccine were classified as coadministered. Serious and non-serious reports were analyzed separately. Focused strata included death and Guillain–Barré syndrome, as well as other clinically important Preferred Terms identified during clinical review.

All analyses were performed using R (version 4.3.2). ROR and PRR with confidence intervals were computed using base R functions and the epitools package (version 0.5-10.1). EBGM estimates were obtained using the openEBGM package (version 0.9.1). BCPNN analyses were implemented in R based on the World Health Organization–Uppsala Monitoring Centre (WHO–UMC) definition of the Information Component and its lower confidence bound.

## 3 Results

### 3.1 Characteristics of VAERS reports

A total of 16,611 adverse event (AE) reports following MenB vaccination were identified in VAERS between 2015 and 2025, with data available through 30 September 2025 (Table 1). Females accounted for 55.8% (n = 8,226) and males for 44.2% (n = 6,508), and age information was available for 11,635 reports. Among these, 23.9% involved children under 2 years, 2.5% were from those aged two to 3 years, 2.2% from those aged three to 5 years, 5.6% from those aged six to 11 years, 39.4% from adolescents aged twelve to 17 years, and 26.3% from individuals 18 years or older.

**TABLE 1:** Baseline characteristics of VAERS reports following Men B vaccination, 2015–2025, with data available through 30 September 2025.

Characteristics of VAERS reports following men B, 2015Q1–2025Q3			
Characteristics	Vaccine-induced AE reports (N = 16,611)		
Number of events	Available number^a^	Case number	Case proportion, %
Gender	**14,734**	​	​
F	​	8,226	55.83%
M	​	6,508	44.17%
Age (years)	**11,635**	​	​
<2 years	​	2,785	23.94%
2 ≤ age < 3 years	​	296	2.54%
3 ≤ age < 6 years	​	259	2.23%
6 ≤ age < 12 years	​	648	5.57%
12 ≤ age < 18 years	​	4,580	39.36%
≥18 years	​	3,067	26.36%
Serious/non-serious status^b^	**16,611**	​	​
Non-serious	​	13,298	80.10%
Serious	​	3,313	19.90%
Outcomes for serious reports	**3954** ^c^	​	​
Death	​	105	2.92%
Hospitalization	​	2,937	81.72%
Life-threatening illness	​	310	8.63%
Prolongation of existing hospitalization	​	28	0.78%
Permanent disability	​	214	5.95%
Vaccine name	16,681^c^	​	​
MENINGOCOCCAL B (BEXSERO)	​	12,704	76.20%
MENINGOCOCCAL B (TRUMENBA)	​	3,977	23.80%
Men B given alone	**16,611**	​	​
Vaccine co-administration	​	5,769	34.70%
Alone (total)	​	10,842	65.30%
TRUMENBA-alone	​	2,399	14.44%
BEXSERO-alone	​	8,443	50.83%
Vaccine dose series	**9,459**	​	​
1	​	6,156	65.08%
2	​	2,723	28.79%
≥3	​	583	6.16%
Onset time	**12,191**	​	​
0–30 days	​	11,857	97.26%
31–60 days	​	124	0.92%
61–90 days	​	38	0.28%
91–120 days	​	26	0.19%
121–150 days	​	18	0.13%
151–180 days	​	9	0.07%
181–360 days	​	41	0.30%
>360 days	​	78	0.58%

^a^ Available number indicates that missing data has been removed.

^b^ Serious reports are those describing death, life-threatening illness, hospitalization or prolongation of existing hospitalization, or permanent disability.

^c^ There exists an ID, that corresponds to multiple records.

Most reports (80.1%) were classified as non-serious, while 19.9% met the FDA criteria for serious outcomes, which included death, life-threatening illness, hospitalization, permanent disability, or congenital anomalies. Hospitalization was the most frequent serious outcome (81.7%), followed by life-threatening illness (8.6%) and death (2.9%).

MenB vaccines were administered alone in 65.3% of reports and co-administered with other vaccines in 34.7%. Bexsero accounted for 76.2% of reports and Trumenba for 23.8%. Most AEs (97.3%) occurred within 30 days of vaccination. The majority of reports were associated with the first dose (65.1%), with fewer related to the second (28.8%) or later doses (6.2%).

**FIGURE 1:**
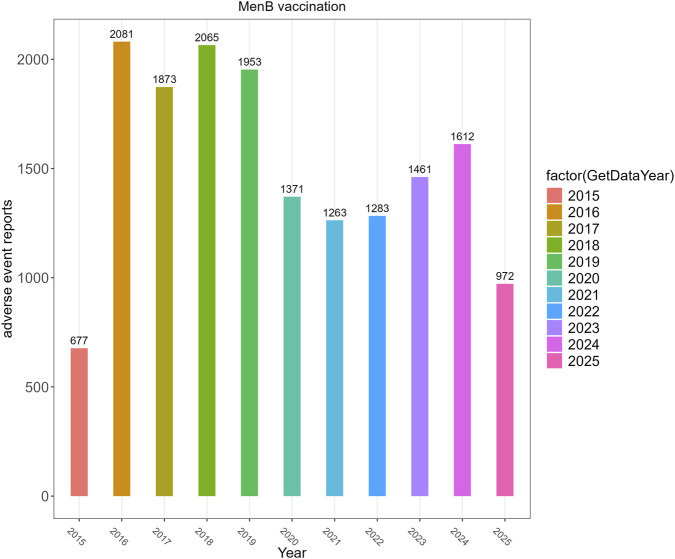
Annual distribution of VAERS reports following meningococcal group B vaccination.

The temporal trend of MenB-related reports (Figure 1) showed a peak from 2016 to 2018, followed by a marked decline during 2020–2021 coinciding with the COVID-19 pandemic, and a gradual rebound thereafter. Because VAERS is a passive reporting system, pandemic period fluctuations were interpreted descriptively as changes in healthcare access and reporting behavior rather than adjusted analytically, and all disproportionality calculations were conducted within the same study window for MenB and non MenB reports. Given that the non-MenB comparator pool included vaccines administered across heterogeneous age groups and clinical indications, the descriptive and disproportionality findings presented below should be interpreted as reporting patterns within a passive surveillance framework rather than as direct estimates of comparative risk.

Age-stratified analysis (Table 2) showed that adolescents aged twelve to 17 years contributed the largest proportion of reports (39.6%), followed by adults 18 years and older (26.3%) and infants under 2 years (23.9%). Serious reports were most frequent among infants (47.7%) and declined progressively with age, reaching 4.3% in adolescents and 4.1% in adults. Deaths were rare overall but occurred disproportionately in infants.

**TABLE 2:** Age-stratified VAERS reports following MenB vaccination, showing the highest number of reports in adolescents (12–18 years), while the proportion of serious outcomes was greatest among infants under 2 years.

Age groups	<2 years	2 ≤ age < 3Y	3 ≤ age < 6 years	6 ≤ age < 12 years	12 ≤ age < 18 years	≥18 years
Gender						
F	1,183 (42.5%)	130 (43.9%)	114 (44.0%)	278 (42.9%)	2,671 (58.3%)	2029 (66.2%)
M	1,336 (48.0%)	137 (46.3%)	118 (45.6%)	335 (51.7%)	1849 (40.4%)	996 (32.5%)
Serious/non-serious status						
Non-serious	1,457 (52.3%)	183 (61.8%)	166 (64.1%)	548 (84.6%)	4,384 (95.7%)	2,941 (95.9%)
Serious	1,328 (47.7%)	113 (38.2%)	93 (35.9%)	100 (15.4%)	196 (4.3%)	126 (4.1%)
MenB vaccine given alone						
Vaccine co-administration	921 (33.1%)	23 (7.8%)	18 (6.9%)	199 (30.7%)	2,307 (50.4%)	942 (30.7%)
Alone	1864 (66.9%)	273 (92.2%)	241 (93.1%)	449 (69.3%)	2,273 (49.6%)	2,125 (69.3%)
Outcomes, n (%)						
Death	29 (1.0%)	2 (0.7%)	3 (1.2%)	4 (0.6%)	2 (0.0%)	6 (0.2%)
Hospitalization	1,208 (43.4%)	106 (35.8%)	133 (2.3%)	92 (14.2%)	167 (3.6%)	96 (3.1%)
Life-threatening illness	128 (4.6%)	4 (1.4%)	78 (30.1%)	4 (0.6%)	25 (0.5%)	26 (0.8%)
Prolongation of existing hospitalization	9 (0.3%)	0	1 (0.4%)	3 (0.5%)	1 (0.0%)	1 (0.0%)
Permanent disability	65 (2.3%)	4 (1.4%)	9 (3.5%)	7 (1.1%)	29 (0.6%)	19 (0.6%)
Recover	1,566 (56.2%)	151 (51.0%)	141 (54.4%)	348 (53.7%)	2,278 (49.7%)	1,353 (44.1%)
Onset time						
0–30 days	2,269	225	213	600	4,046	2,667
31–60 days	20	4	4	7	19	20
61–90 days	7	1	0	0	8	3
91–120 days	3	2	0	0	6	3
121–150 days	1	1	0	0	1	6
151–180 days	0	0	0	1	0	0
181–360 days	7	1	2	4	5	3
>360 days	3	1	2	6	12	10

### 3.2 Distribution of reported adverse events

**FIGURE 2:**
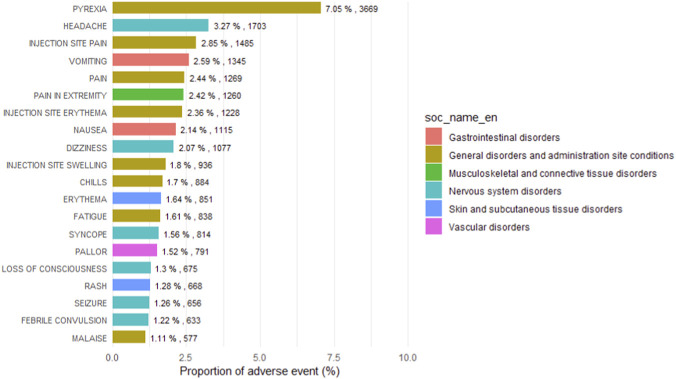
Distribution of the most frequently reported adverse events after MenB vaccination.

**FIGURE 3:**
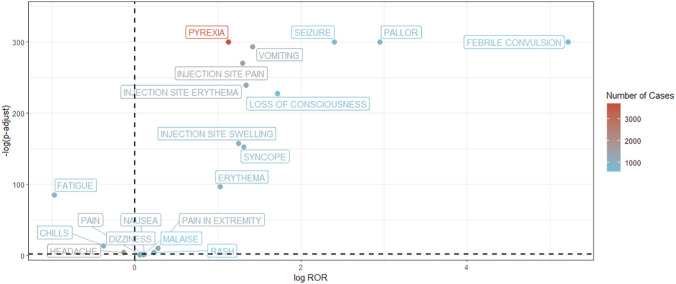
Volcano plot of Preferred Terms following MenB vaccination displaying reporting odds ratios and corresponding p values.

MenB-related reports encompassed a broad spectrum of clinical manifestations, showing distinct clustering at both the Preferred Term and System Organ Class levels (Figure 2 **)**. Events under general disorders and administration site conditions formed the largest category, including pyrexia (7.05%), injection site pain (2.85%), and injection site erythema (2.36%). Neurological events contributed substantially, with headache (3.27%), dizziness (2.07%), syncope (1.56%), seizure (1.26%), and febrile convulsion (1.22%) being recurrently reported. Gastrointestinal events such as vomiting (2.59%) and nausea (2.14%) were also frequent, while cutaneous reactions including erythema (1.64%) and rash (1.28%) were notable (Figure 3).

Many of these frequently reported events align with the known safety profiles of Bexsero and Trumenba documented by the U.S. Food and Drug Administration and European Medicines Agency. Reactions such as pyrexia, injection site pain, erythema, headache, fatigue, and myalgia are listed as common in their labeling, and similar patterns were reported in previous studies. Their recurrence in the present dataset therefore likely reflects expected reactogenicity rather than new safety signals. Nonetheless, several terms with elevated reporting odds ratios were not prominently listed in product labeling, including loss of consciousness, pallor, and selected hematologic or vascular events. Although their absolute numbers were low, their disproportionality suggests they may warrant further assessment as summarized in Table 3, with the contingency table structure and signal detection criteria provided in Supplementary Table S1 and the false discovery rate–adjusted results presented in Supplementary Table S2. After Benjamini Hochberg adjustment, the majority of PTs highlighted in Table 3 retained statistical support, whereas a small subset did not meet the FDR <0.05 threshold and is listed in Supplementary Table S3, including lymphadenitis, circulatory collapse, urinary incontinence, and jaundice.

**TABLE 3:** Disproportionality analysis of adverse events after MenB vaccination across System Organ Classes.

SOC	PT (preferred term)	N	ROR (95%Cl)	IC (IC025)	PRR (χ^2^)	EBGM (EBGM05)
Blood and lymphatic system disorders	Thrombocytopenia^a^	49	1.48 (1.12–1.97)	0.56 (0.15)	1.48 (7.63)	1.48 (1.17)
	Leukocytosis^a^	37	3.26 (2.36–4.52)	1.68 (1.21)	3.26 (56.75)	3.21 (2.45)
	Neutropenia^a^	28	5.25 (3.6–7.65)	2.35 (1.81)	5.25 (92.96)	5.1 (3.72)
	Leukopenia	20	3.85 (2.47–6)	1.92 (1.28)	3.85 (41.07)	3.77 (2.6)
	Lymphadenitis^a^	18	1.72 (1.08–2.74)	0.78 (0.11)	1.72 (5.37)	1.71 (1.16)
Cardiac disorders	Bradycardia^a^	44	1.79 (1.33–2.41)	0.83 (0.4)	1.79 (15.22)	1.78 (1.39)
	Coronary artery dilatation^a^	4	11.73 (4.23–32.47)	3.45 (2.1)	11.73 (36.34)	10.93 (4.66)
	Bradycardia neonatal^a^	3	21.99 (6.53–73.99)	4.27 (2.71)	21.99 (52.26)	19.25 (6.97)
Ear and labyrinth disorders	Ear swelling^a^	16	2.35 (1.44–3.86)	1.22 (0.52)	2.35 (12.24)	2.33 (1.54)
	Tympanic membrane hyperaemia	6	21.99 (9.32–51.86)	4.27 (3.1)	21.99 (104.51)	19.25 (9.39)
Eye disorders	Eye movement disorder^a^	128	6.25 (5.23–7.46)	2.59 (2.33)	6.24 (539.83)	6.02 (5.19)
	Gaze palsy^a^	100	13.27 (10.81–16.29)	3.61 (3.31)	13.25 (1,038.99)	12.24 (10.31)
	Photophobia^a^	66	1.76 (1.38–2.25)	0.81 (0.46)	1.76 (21.56)	1.75 (1.43)
	Eyelid oedema	36	5.71 (4.09–7.97)	2.47 (1.98)	5.71 (134.65)	5.53 (4.19)
	Blindness^a^	34	1.55 (1.11–2.18)	0.63 (0.14)	1.55 (6.59)	1.55 (1.16)
Gastrointestinal disorders	Vomiting	1,345	2.67 (2.53–2.82)	1.38 (1.3)	2.63 (1,347.93)	2.6 (2.49)
	Nausea	1,115	1.08 (1.02–1.15)	0.11 (0.02)	1.08 (6.45)	1.08 (1.03)
	Diarrhoea^a^	471	1.2 (1.09–1.31)	0.26 (0.12)	1.2 (15.14)	1.19 (1.11)
	Haematochezia^a^	84	3.34 (2.69–4.15)	1.72 (1.4)	3.34 (134.38)	3.28 (2.74)
	Lip swelling^a^	83	1.3 (1.04–1.61)	0.37 (0.05)	1.3 (5.55)	1.29 (1.08)
General disorders and administration site conditions	Pyrexia	3,669	2.18 (2.11–2.25)	1.07 (1.02)	2.11 (2,177.55)	2.1 (2.04)
	Injection site pain	1,485	2.46 (2.34–2.59)	1.27 (1.19)	2.43 (1,238.49)	2.4 (2.3)
	Injection site erythema	1,228	2.53 (2.39–2.68)	1.31 (1.23)	2.5 (1,097.85)	2.48 (2.36)
	Injection site swelling	936	2.37 (2.22–2.53)	1.22 (1.13)	2.35 (721.2)	2.33 (2.21)
	Crying	559	10.84 (9.95–11.82)	3.33 (3.21)	10.76 (4,613.37)	10.09 (9.39)
Hepatobiliary disorders	Jaundice^a^	10	1.89 (1.01–3.52)	0.91 (0.03)	1.89 (4.13)	1.88 (1.11)
Immune system disorders	Hypersensitivity	127	1.23 (1.03–1.47)	0.3 (0.04)	1.23 (5.43)	1.23 (1.06)
	Milk allergy^a^	8	5.86 (2.89–11.89)	2.5 (1.52)	5.86 (31.03)	5.68 (3.14)
	Type III immune complex mediated reaction	7	3.64 (1.72–7.7)	1.84 (0.8)	3.64 (13.07)	3.57 (1.91)
	Hypogammaglobulinaemia^a^	4	4.48 (1.65–12.1)	2.13 (0.81)	4.48 (10.48)	4.37 (1.9)
Infections and Infestations	Cellulitis	162	3 (2.57–3.5)	1.56 (1.33)	2.99 (210.89)	2.95 (2.59)
	Meningococcal infection^a^	121	71.36 (57.42–88.68)	5.59 (5.29)	71.23 (5,638.4)	48.26 (40.24)
	Injection site cellulitis	87	5.86 (4.73–7.27)	2.5 (2.19)	5.86 (337.07)	5.67 (4.74)
	Meningitis^a^	70	12.27 (9.61–15.66)	3.51 (3.15)	12.26 (667.98)	11.39 (9.29)
	Viral infection	55	4.9 (3.75–6.41)	2.25 (1.86)	4.9 (165.11)	4.77 (3.81)
Metabolism and nutrition disorders	Decreased appetite^a^	302	1.51 (1.35–1.69)	0.58 (0.42)	1.51 (50.83)	1.5 (1.36)
	Poor feeding Infant^a^	70	28.77 (22.26–37.18)	4.6 (4.23)	28.74 (1,567.03)	24.19 (19.52)
	Dehydration^a^	62	1.47 (1.15–1.89)	0.55 (0.19)	1.47 (9.34)	1.47 (1.19)
	Hypophagia^a^	35	2.21 (1.58–3.08)	1.13 (0.65)	2.21 (22.78)	2.19 (1.66)
	Diet refusal^a^	22	7.96 (5.19–12.23)	2.93 (2.31)	7.96 (127.03)	7.6 (5.31)
Musculoskeletal and connective tissue disorders	Pain in extremity^a^	1,260	1.21 (1.14–1.28)	0.27 (0.18)	1.2 (43.74)	1.2 (1.15)
	Mobility decreased^a^	262	1.6 (1.42–1.81)	0.67 (0.49)	1.6 (58.55)	1.59 (1.44)
	Musculoskeletal stiffness^a^	236	1.97 (1.73–2.24)	0.97 (0.78)	1.97 (110.96)	1.95 (1.75)
	Neck pain^a^	223	1.36 (1.19–1.55)	0.44 (0.25)	1.36 (21.11)	1.36 (1.21)
	Posture abnormal^a^	74	7.86 (6.22–9.93)	2.91 (2.57)	7.85 (419.79)	7.5 (6.17)
Nervous system disorders	Syncope	814	2.48 (2.31–2.66)	1.29 (1.18)	2.46 (699.52)	2.44 (2.3)
	Loss of consciousness^a^	675	3.29 (3.05–3.56)	1.69 (1.57)	3.27 (1,043.18)	3.22 (3.02)
	Seizure^a^	656	5.28 (4.88–5.71)	2.35 (2.23)	5.24 (2,175.15)	5.09 (4.77)
	Febrile convulsion^a^	633	37.16 (34.04–40.55)	4.89 (4.76)	36.8 (17,628.39)	29.62 (27.53)
	Tremor^a^	429	2.04 (1.85–2.24)	1.01 (0.87)	2.03 (221.28)	2.01 (1.86)
Psychiatric disorders	Irritability^a^	270	7.21 (6.38–8.15)	2.79 (2.61)	7.19 (1,371.79)	6.9 (6.23)
	Restlessness^a^	135	5.1 (4.29–6.05)	2.31 (2.06)	5.09 (428.72)	4.95 (4.29)
	Staring^a^	61	9.53 (7.35–12.35)	3.17 (2.79)	9.52 (436.9)	9 (7.25)
	Apathy	57	6.08 (4.66–7.92)	2.55 (2.17)	6.07 (231.9)	5.87 (4.7)
	Abnormal behaviour^a^	56	5.03 (3.86–6.57)	2.29 (1.9)	5.03 (174.82)	4.9 (3.92)
Renal and urinary disorders	Urinary incontinence^a^	28	1.51 (1.04–2.19)	0.59 (0.05)	1.51 (4.78)	1.51 (1.1)
	Chromaturia^a^	21	2.12 (1.38–3.26)	1.07 (0.45)	2.12 (12.22)	2.1 (1.47)
	Nephrotic syndrome^a^	10	2.21 (1.19–4.13)	1.13 (0.26)	2.21 (6.56)	2.2 (1.3)
	Haemoglobinuria^a^	5	40.72 (15.12–109.67)	5 (3.69)	40.71 (151.59)	32.08 (14)
	Anuria^a^	4	3.39 (1.26–9.13)	1.74 (0.43)	3.39 (6.58)	3.33 (1.46)
Reproductive system and breast disorders	Scrotal swelling^a^	3	4.89 (1.55–15.44)	2.25 (0.78)	4.89 (8.97)	4.76 (1.82)
Respiratory, thoracic and mediastinal disorders	Apnoea^a^	105	12.43 (10.18–15.17)	3.53 (3.23)	12.41 (1,015.79)	11.52 (9.75)
	Respiratory arrest^a^	48	4.84 (3.63–6.45)	2.24 (1.82)	4.84 (141.38)	4.71 (3.7)
	Tachypnoea^a^	32	2.37 (1.67–3.36)	1.23 (0.72)	2.37 (24.91)	2.35 (1.75)
	Hypopnoea^a^	31	3.67 (2.57–5.24)	1.85 (1.33)	3.67 (58.76)	3.6 (2.68)
	Pharyngeal erythema^a^	22	4.56 (2.98–6.97)	2.15 (1.54)	4.56 (59.32)	4.45 (3.12)
Skin and subcutaneous tissue disorders	Erythema^a^	851	2.04 (1.91–2.18)	1.01 (0.91)	2.03 (439.68)	2.01 (1.9)
	Rash	668	1.17 (1.09–1.27)	0.23 (0.11)	1.17 (16.89)	1.17 (1.1)
	Urticaria^a^	545	1.67 (1.54–1.82)	0.73 (0.61)	1.67 (144.54)	1.66 (1.55)
	Skin warm	295	2.09 (1.86–2.35)	1.05 (0.88)	2.09 (164.77)	2.07 (1.88)
	Petechiae^a^	128	6.06 (5.07–7.23)	2.55 (2.29)	6.05 (517.9)	5.85 (5.04)
Vascular disorders	Pallor^a^	791	7.71 (7.17–8.28)	2.87 (2.76)	7.63 (4,335.24)	7.3 (6.87)
	Cyanosis^a^	241	10.33 (9.06–11.77)	3.28 (3.08)	10.29 (1889.97)	9.68 (8.68)
	Kawasaki’s disease^a^	113	37.88 (30.8–46.59)	4.92 (4.62)	37.81 (3,219.6)	30.26 (25.45)
	Peripheral coldness^a^	45	1.45 (1.08–1.94)	0.53 (0.1)	1.45 (6.15)	1.44 (1.13)
	Circulatory collapse^a^	27	1.53 (1.05–2.23)	0.61 (0.06)	1.53 (4.88)	1.52 (1.11)

^a^ Preferred Terms not identified in the FDA- or EMA-approved product labeling for MenB vaccines.

### 3.3 Signal detection results

**FIGURE 4:**
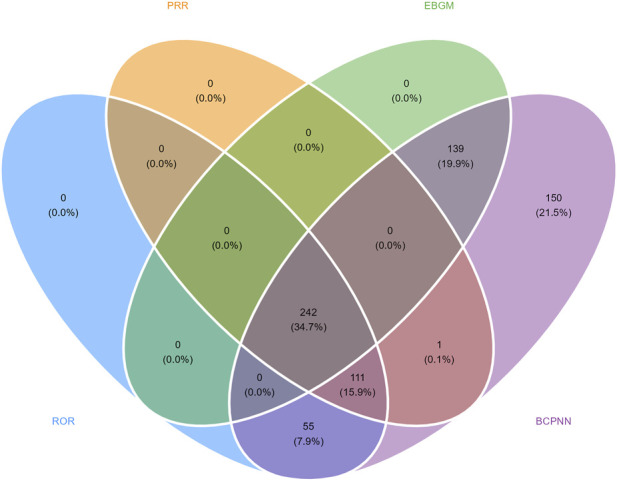
Venn diagram showing overlap of signal detection methods.

The integrated disproportionality analysis using ROR, PRR, EBGM and BCPNN identified a large number of potential signals (Figure 4). A total of 242 PTs (34.7%) was detected by all four methods, representing the most robust set with consistent statistical support. This overlapping set included both expected reactogenic events, such as pyrexia and injection site pain, and several unlabeled neurological manifestations of potential clinical relevance, including hypotonia, pallor, and loss of consciousness. An additional 111 PTs (15.9%) were detected by three methods. BCPNN uniquely identified 150 PTs (21.5%), and 139 PTs (19.9%) were detected jointly by BCPNN and EBGM. PTs detected exclusively by BCPNN were typically very low-frequency terms, often supported by fewer than ten reports, such as developmental speech disorder, yellow skin and granuloma, illustrating the capacity of the Bayesian framework to capture sparse and rare event profiles that may not reach statistical thresholds under frequentist disproportionality metrics. Smaller overlaps were seen between BCPNN and ROR (55 PTs, 7.9%) and between BCPNN and PRR (1 PT, 0.1%).

When stratified by vaccine product, the distribution of statistically supported PT signals showed observable heterogeneity between Bexsero and Trumenba, as detailed in Supplementary Table S4 and Supplementary Table S5. Certain hematologic and immune-related PTs contributing to the overall MenB signal profile were predominantly represented in reports associated with Bexsero, whereas several neurologic or ocular PTs were more frequently detected in Trumenba-associated reports. These findings indicate that the aggregated MenB signal spectrum presented in Table 3 reflects a composite of product-specific reporting patterns rather than a fully uniform class-wide distribution.

However, due to VAERS is a spontaneous reporting system and the comparator pool encompasses heterogeneous vaccine recipients, these observations should not be interpreted as evidence of comparative safety differences between products.

### 3.4 Subgroup and temporal patterns

**FIGURE 5:**
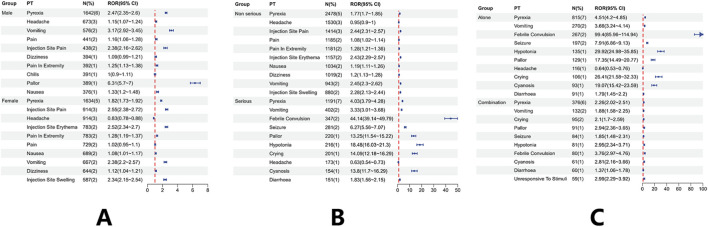
**(A)** Sex-stratified analysis showing higher signals for pallor and vomiting in males and for local reactions in females. **(B)** Serious reports were enriched for febrile convulsion, seizure, hypotonia, pallor, and cyanosis, while non-serious reports mainly involved mild local or systemic events. **(C)** Severe adverse events were more frequent when meningococcal group B vaccines were given alone rather than with other vaccines.

Sex-stratified disproportionality analysis revealed distinct patterns in the reporting of specific Preferred Terms (PTs) (Figure 5A). In males, pallor showed the strongest association with a markedly elevated reporting odds ratio (ROR), while vomiting and pyrexia also demonstrated significant disproportionality. In females, local reactions such as injection site pain, erythema, and swelling were more prominent, alongside moderately increased reporting of vomiting. These sex-based differences persisted despite broadly similar baseline age distributions between male and female recipients (Table 2).

Comparisons between serious and non-serious reports indicated that several systemic manifestations were disproportionately enriched among serious outcomes (Figure 5B **)**. Febrile convulsion, seizure, hypotonia, pallor, cyanosis, and persistent crying exhibited notably elevated RORs in serious reports, whereas they were rare among non-serious reports. Conversely, non-serious reports were dominated by mild local or transient systemic reactions such as injection site pain, nausea, dizziness, and headache, which showed minimal or no disproportionality in serious reports.

Analysis stratified by vaccination mode showed that several severe PTs were primarily reported when MenB vaccines were administered alone (Figure 5C **)**. Febrile convulsion, hypotonia, pallor, cyanosis, and seizure had substantially higher disproportionality when MenB was given without concomitant vaccines, while coadministration was associated with attenuated RORs for these outcomes. This pattern may reflect differences in recipient characteristics or reporting practices across vaccination settings.

**FIGURE 6:**
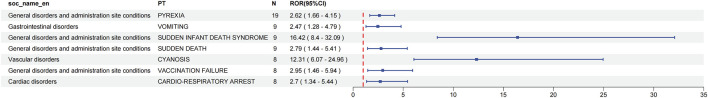
Disproportional reporting patterns of fatal adverse event Preferred Terms following MenB vaccination in VAERS. *VAERS is a passive surveillance system and does not permit causal inference. Infection-related Preferred Terms may reflect breakthrough disease, background incidence in high-risk populations, or diagnostic attribution within spontaneous reports rather than vaccine-attributable toxicity.

Evaluation of death-associated reports identified several PTs with strong disproportionality signals across multiple System Organ Classes (Figure 6). Sudden infant death syndrome, meningococcal sepsis, meningococcal infection, and meningococcal bacteraemia all showed extremely high ROR values and were concurrently detected by PRR, EBGM, and Bayesian methods. These events clustered within the infections and general disorders SOCs, representing a small but distinct subset of fatal outcomes with specific clinical presentations. Importantly, VAERS data do not permit causal attribution, and disproportional reporting of infection-related PTs does not establish that vaccination precipitated meningococcal disease. Such reports may reflect breakthrough infections, infections occurring before protective immunity was established, background disease incidence in high-risk populations, or diagnostic and reporting attribution within a passive surveillance system. Therefore, these findings should be interpreted strictly as reporting signals within VAERS and not as indicators of vaccine-attributable infection or reduced vaccine effectiveness. Because these infection-related PTs may also reflect underlying breakthrough disease or diagnostic attribution within passive surveillance reports, they were interpreted descriptively within the VAERS reporting context rather than as direct indicators of vaccine-attributable toxicity.

**FIGURE 7:**
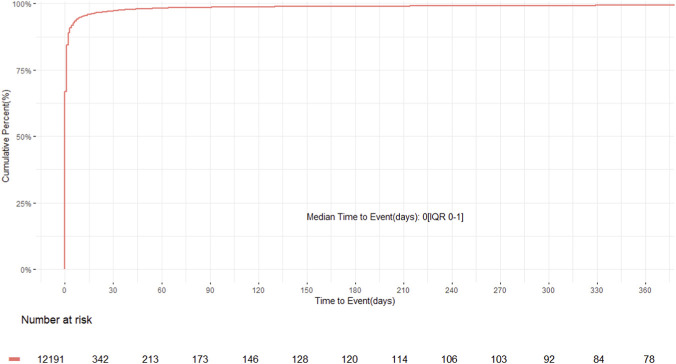
Cumulative distribution of time to onset for adverse events following MenB vaccination, showing that nearly all events occurred within the first day after vaccination, with a median onset time of 0 days reflecting the high proportion of same-day onset reports.

Time-to-onset analysis showed that most adverse events occurred shortly after vaccination. The cumulative incidence curve (Figure 7) demonstrated a steep early rise, with a median onset of 0 days (interquartile range, IQR 0–1), indicating that nearly all events emerged within the first few days post-immunization. This pattern was consistent across all six age groups (Supplementary Figure S1), each showing a median onset of 0 days (IQR 0–2), suggesting that age had minimal influence on the latency of adverse event occurrence. Sex-stratified analysis (Supplementary Figure S2) revealed a subtle but statistically significant difference, with females experiencing slightly earlier onset than males (p < 0.001), although the absolute difference was minimal. Comparison of MenB administered alone versus coadministered with other vaccines (Supplementary Figure S3) showed a modest difference (p = 0.03), with coadministration associated with a slightly more concentrated early onset pattern. Finally, serious events occurred more rapidly than non-serious events (Supplementary Figure S4, p < 0.001). The cumulative curve for serious events rose more steeply, indicating that severe outcomes tended to appear soon after vaccination.

## 4 Discussion

This analysis draws on the largest available post-licensure VAERS dataset for MenB vaccines, including MenB-4C and MenB-FHbp, covering multiple years of routine use. Across several thousand reports, most adverse events were mild and consistent with pre-licensure reactogenicity: local injection-site reactions, fever, headache, and other mild systemic symptoms. Serious events were rare, and no new clusters or unexpected demographic concentrations emerged. The present study relied on disproportionality analysis within a heterogeneous non-MenB comparator pool in a passive surveillance system, and the findings should therefore be interpreted as patterns of reporting imbalance rather than as direct estimates of relative or absolute risk.

When interpreted in the context of prior research, the observed patterns closely align with earlier post-marketing assessments, which reported frequent local and systemic reactions but few serious outcomes (Duffy et al., 2020; Perez-Vilar et al., 2022). Those studies were limited to the early post-licensure years and included fewer than two thousand reports each, constraining their ability to evaluate rare events or temporal changes. In contrast, the present analysis spans 2015 through 2025 and captures the transition from outbreak-targeted use to routine adolescent immunization, as well as the disruptions caused by the COVID-19 pandemic. The persistence of similar adverse event profiles across these programmatic shifts is consistent with a broadly similar reporting pattern over time, despite changes in target populations, coadministration practices, and healthcare delivery. Taken together, these findings indicate that no new or unexpected safety signals of substantial magnitude emerged during a decade of post-licensure use, within the constraints inherent to spontaneous reporting data and heterogeneous background reporting rates. Within the methodological limits of passive surveillance, these observations are consistent with a stable reporting profile over time rather than evidence of newly emerging safety concerns.

The biological plausibility of the signals supports their interpretation as manifestations of vaccine reactogenicity rather than unexpected toxicities. MenB vaccines, formulated with recombinant antigens and outer-membrane vesicles, engage innate immunity through pattern recognition receptors including Toll-like receptors, which trigger rapid release of pro-inflammatory cytokines such as IL-6 and IL-1β. These mediators account for common systemic manifestations including fever and malaise, while local swelling and pain reflect cytokine-driven recruitment of neutrophils and activation of complement at the injection site. Autonomic reactions such as syncope are thought to result from acute vascular and neuroendocrine responses to peripheral inflammation (Deghmane and Taha, 2022; Valente et al., 2020; O'Ryan et al., 2014). Even within the small subset of serious reports, such as seizures, hypotonia, or apnea, similar phenomena have been described following pediatric and adolescent vaccines and are widely attributed to fever-mediated neuronal excitability or transient systemic stress rather than direct neurotoxic effects. Importantly, no enrichment of autoimmune, demyelinating, or thromboinflammatory conditions was identified in the present analysis, which argues against vaccine-specific pathogenic mechanisms, although the absence of disproportional reporting does not exclude extremely rare associations that would require alternative study designs for detection (Zahid et al., 2025).

Subgroup analyses showed modest heterogeneity. Although males and females had broadly similar distributions of adverse event Preferred Terms, syncope and similar autonomic reactions appeared somewhat more frequent among females, aligning with known sex-based differences in vasovagal responses. Serious event reports occurred more often among infants and toddlers in our data, which may reflect both their physiological vulnerability and greater reporting vigilance in that age group. Reports involving coadministration with other vaccines constituted a substantial fraction of total MenB reports, but in our analyses, we did not detect new signals arising uniquely from coadministration, and differences in disproportionality metrics were minimal. Apparent enrichment of certain severe events when MenB vaccines were administered alone should be interpreted cautiously, as this pattern is likely influenced by residual confounding by age, given that infants and young children are both more susceptible to events such as febrile convulsions and more likely to receive MenB without concomitant vaccines under specific risk-based recommendations. Death-associated reports were rare, involving meningococcal sepsis, bacteraemia, or cases labeled SIDS in a few instances; given their low numbers and heterogeneous clinical details, it is difficult to infer causality, underscoring the need for detailed case-level review (Moro et al., 2015). Moreover, VAERS does not consistently capture sufficient clinical information to characterize fatal infectious outcomes in detail, including confirmation of diagnosis, cause of death, or relevant comorbid conditions, precluding systematic case-level summarization. Disproportional reporting of infection-related Preferred Terms in this context does not establish that vaccination precipitated meningococcal disease. Within a passive surveillance system such as VAERS, such reports may reflect breakthrough infections occurring despite prior immunization, infections that developed before protective immunity was established, background incidence in high-risk populations, or inaccuracies in diagnostic attribution within spontaneous reports. In the absence of verified denominator data and structured case validation, these observations cannot be used to evaluate vaccine effectiveness or to infer causality from temporally associated events.

In addition, product-stratified signal evaluation indicated that the overall MenB disproportionality spectrum reflected a composite of vaccine-specific reporting patterns rather than a completely uniform class-wide distribution, with certain hematologic or immune-related terms contributing predominantly within one product while selected neurologic or ocular manifestations appeared relatively more prominent in the other. This observation should be interpreted descriptively within the context of passive surveillance and does not imply differential causal risk between products. Given the absence of denominator data and the heterogeneous comparator population, any apparent product-level differences should be regarded as exploratory reporting patterns rather than comparative safety estimates. VAERS data are not suitable for formal comparative safety analyses between Bexsero and Trumenba, as differences in age distribution, clinical indication, and reporting behavior cannot be adequately controlled within a passive surveillance framework. The observed heterogeneity may nevertheless reflect several non-mutually exclusive mechanisms that warrant further investigation. The presence of outer membrane vesicles in Bexsero, which contain relatively higher lipopolysaccharide content, could plausibly provoke a more pronounced innate immune or hematologic acute-phase response compared with the recombinant protein and alum-based formulation of Trumenba. Alternatively, differential use patterns across age groups, including Bexsero’s incorporation into infant risk-based schedules and Trumenba’s earlier focus on adolescents, may generate distinct background susceptibilities to events such as febrile convulsion in infancy or syncope in adolescence. Disentangling intrinsic product effects from confounding by indication, age distribution, and reporting context will require controlled observational or active-surveillance studies specifically designed for comparative safety evaluation.

Time-to-onset analyses showed that most events occurred on day 0, with a sharp decline thereafter. This pattern was observed consistently across sex, age, seriousness, and coadministration strata, indicating broadly similar latencies among subgroups. The early clustering mirrors the reactogenicity window described in clinical trials and mechanistic studies, in which solicited local and systemic symptoms typically emerge within the first 24–48 h and resolve shortly afterward (Ostergaard et al., 2017). This temporal concentration is biologically plausible, as vaccine antigens and adjuvant components rapidly trigger innate immune sensors, leading to transient cytokine release, complement activation, and autonomic responses such as fever or syncope. The acute kinetics reflect the short-lived nature of these pathways rather than delayed toxicities. Although small statistical differences were detectable, such as slightly longer median onset after coadministration, these shifts are more likely attributable to overlapping inflammatory responses from multiple vaccines than to any distinct mechanistic process (Diaz-Gonzalez et al., 2023). Collectively, these data suggest that counseling and surveillance should focus on the first one to 2 days post-vaccination, the period of greatest likelihood for observable events and lowest probability for late-onset sequelae (Herve et al., 2019).

Beyond expected reactogenicity, several Preferred Terms exhibiting elevated disproportionality were not included in the FDA- or EMA-approved product information for either MenB vaccine. Although these unlabeled signals were infrequent in absolute number, they demonstrated consistent statistical enrichment across multiple signal detection approaches and encompassed clinical features such as pallor, loss of consciousness, febrile convulsion, hypotonia, cyanosis, and selected hematologic or vascular manifestations. Importantly, stratified analyses indicated that the distribution of these signals was not entirely uniform across vaccine products, suggesting that the aggregated MenB safety profile may reflect partially overlapping yet distinct reporting patterns rather than a single homogeneous biological effect.

From a clinical perspective, several of these manifestations overlap with autonomic or vasovagal phenomena and age-related physiological vulnerability in infancy, and the constellation of hypotonia, pallor, reduced responsiveness, and cyanosis is also consistent with features described in hypotonic-hyporesponsive episodes, a rare but recognized post-vaccination event in early childhood that is typically transient and not associated with persistent neurological sequelae (DuVernoy et al., 2000; Vigo et al., 2017). However, VAERS reports generally lack sufficient detail regarding duration, recovery course, and alternative diagnoses, precluding formal adjudication of hypotonic-hyporesponsive episodes and limiting the ability to distinguish such presentations from syncope, post-ictal states, or other transient physiological responses. Accordingly, the present findings should not be interpreted as confirmation of hypotonic-hyporesponsive episodes but rather as reporting patterns that share partial clinical overlap with such syndromes. In the absence of sufficient clinical detail within VAERS to permit formal adjudication, these observations should therefore be interpreted cautiously as hypothesis-generating rather than indicative of novel vaccine-specific toxicity. Furthermore, evaluation of PTs losing statistical support after false discovery rate adjustment revealed a heterogeneous composition that included both common or non-specific clinical manifestations and serious events lacking clear biological plausibility for vaccine causation, which collectively reduces the likelihood that FDR-negative findings represent previously unrecognized vaccine-attributable safety signals and supports prioritizing multi-method–consistent, statistically robust signals for focused follow-up in active surveillance or pharmacoepidemiologic studies. Several clinically salient signals, including pallor, hypotonia, and febrile convulsion, retained statistical support after false discovery rate adjustment, reinforcing their robustness against multiple-testing effects and supporting their prioritization for confirmatory evaluation in active surveillance frameworks.

These results have several implications for clinicians, immunization programs, and regulators. For clinicians, the concentration of adverse events within the first 24 h highlights the importance of counseling and close observation during this period, particularly for infants and for adolescents who are receiving MenB vaccines for the first time. Providing anticipatory guidance on common and transient reactions can help reduce anxiety among recipients and may decrease the number of unnecessary medical evaluations. For vaccination programs, the absence of newly emerging reporting imbalances of substantial magnitude in this dataset provides contextual information for ongoing routine adolescent immunization with MenB vaccines, while recognizing the inherent interpretative limits of passive surveillance. Programs should also remain aware that reporting patterns may fluctuate when health services are strained, which should be taken into account when interpreting temporal trends. For regulators, the identification of neurological and dermatological adverse event terms that are not currently included in the product label, yet show consistent disproportionality signals, points to the need for detailed case-level review and the potential integration of active surveillance systems to clarify whether these associations are coincidental or reflect a possible causal relationship (Abitbol et al., 2023; Bonanni et al., 2024).

This study has several strengths. It is the largest MenB vaccine safety evaluation to date, incorporating over 16,000 reports spanning 10 years of post-licensure experience. The extended observation window enabled assessment of temporal shifts in reporting, including during the pandemic period, and provided sufficient exposure to detect rare events. The use of multiple complementary signal detection methods reduces reliance on the assumptions of any single approach, and stratified analyses by age, sex, seriousness, and coadministration status enhance interpretability and contextualization of the signals identified.

Nonetheless, there are several limitations. VAERS is a passive surveillance system, so many adverse events go unreported and submissions often vary in completeness and accuracy (Moro et al., 2019). Media attention or litigation can stimulate reporting in biased ways. Because reliable data on all vaccinated individuals are not available, it is impossible to compute true incidence rates; disproportionality analyses therefore reflect reporting imbalances rather than true incidence or causal risk estimates. Temporal associations do not establish causality, especially when many reports lack sufficient clinical details for adjudication. Although multiple signal detection algorithms were applied and false discovery rate adjustment was used as a secondary robustness assessment, the possibility of chance findings cannot be fully excluded. In addition, the non-MenB comparator consisted of all other vaccines reported to VAERS during the same period, representing a heterogeneous mixture of products with substantially different target populations, background risks, and safety profiles. Such heterogeneity may influence the magnitude and direction of disproportionality estimates and therefore constitutes an important source of residual confounding when interpreting detected signals. In particular, age-related differences between MenB recipients and the heterogeneous non-MenB comparator population may influence both the magnitude and the direction of ROR estimates. Accordingly, the reported RORs and related metrics should not be interpreted as causal or directly comparable risk measures but as indicators of reporting imbalance within the VAERS database. Furthermore, the heterogeneity observed between vaccine-specific signal profiles may reflect differences in reporting behavior, population characteristics, or clinical context rather than intrinsic biological divergence, and therefore should be interpreted cautiously. Finally, residual confounding due to coadministered vaccines, preexisting health conditions or healthcare-seeking behavior may affect our findings in ways that cannot be fully controlled (Cutroneo et al., 2024).

Future investigations should combine passive surveillance data with active monitoring systems and clinical cohorts to allow a more robust assessment of causality. Linking VAERS reports with electronic health records or immunization registries would provide reliable denominators for incidence calculations and create opportunities for longitudinal follow-up of outcomes of interest. Focused pharmacoepidemiologic studies are warranted to evaluate specific unlabeled signals identified here, particularly autonomic and neurologic events in infants, and to further investigate reports of meningococcal disease following vaccination to distinguish temporal coincidence from potential vaccine failure. As MenB vaccines become further integrated into routine immunization programs worldwide, sustained and coordinated safety monitoring that applies harmonized analytic frameworks will be critical for maintaining public trust and supporting ongoing evaluation of safety and benefit within appropriate epidemiologic designs.

## 5 Conclusion

This decade-long analysis of VAERS data provides the most comprehensive post-licensure safety evaluation of meningococcal serogroup B vaccines in the United States. Most reported adverse events were consistent with expected reactogenicity, while serious outcomes were uncommon and did not reveal novel syndromic patterns. The clustering of events within the first few days after vaccination supports a predictable temporal profile. Although several unlabeled Preferred Terms showed disproportionality, their clinical significance remains uncertain. Overall, these findings reaffirm the favorable safety profile of meningococcal serogroup B vaccines while underscoring the importance of continued pharmacovigilance to detect rare or emerging signals.

## Data Availability

Publicly available datasets were analyzed in this study. This data can be found here: https://vaers.hhs.gov/data/datasets.html.

## Supplementary material

SUPPLEMENTARY FIGURE S1 Cumulative distribution of time to onset of adverse events after MenB vaccination by age group, showing that nearly all events occurred within one day across all age categories.

SUPPLEMENTARY FIGURE S2 Cumulative distribution of adverse event onset following MenB vaccination by sex, indicating that both males and females experienced nearly all events within the first day after vaccination with minimal difference between groups.

SUPPLEMENTARY FIGURE S3 Cumulative distribution of time to adverse event onset after MenB vaccination, comparing single and co-administered doses, showing slightly earlier onset in the combination group.

SUPPLEMENTARY FIGURE S4 Cumulative distribution of time to onset for serious and non-serious adverse events after MenB vaccination.

## References

[B1] Abitbol V. Sohn W. Y. Horn M. Safadi M. (2023). Safety and immunogenicity of co-administered meningococcal serogroup B (4CMenB) vaccine: a literature review. Hum. Vaccin Immunother. 19 (2), 2245705. 10.1080/21645515.2023.2245705 37642229 PMC10467517

[B2] Bartoloni A. Norelli F. Ceccarini C. Rappuoli R. Costantino P. (1995). Immunogenicity of meningococcal B polysaccharide conjugated to tetanus toxoid or CRM197 *via* adipic acid dihydrazide. Vaccine 13 (5), 463–470. 10.1016/0264-410x(94)00007-a 7543714

[B3] Bate A. Evans S. J. (2009). Quantitative signal detection using spontaneous ADR reporting. Pharmacoepidemiol Drug Saf. 18 (6), 427–436. 10.1002/pds.1742 19358225

[B4] Bonanni P. Castagna S. Gabutti G. Giuffrida S. Marchetti F. Russo R. (2024). Available evidence on the co-administration of the four-component meningococcal B vaccine (4CMenB) with three vaccines at the same visit among pediatric individuals. Hum. Vaccin Immunother. 20 (1), 2333106. 10.1080/21645515.2024.2333106 38566502 PMC10993916

[B5] Candore G. Juhlin K. Manlik K. Thakrar B. Quarcoo N. Seabroke S. (2015). Comparison of statistical signal detection methods within and across spontaneous reporting databases. Drug Saf. 38 (6), 577–587. 10.1007/s40264-015-0289-5 25899605

[B6] Cutroneo P. M. Sartori D. Tuccori M. Crisafulli S. Battini V. Carnovale C. (2024). Conducting and interpreting disproportionality analyses derived from spontaneous reporting systems. Front. Drug Saf. Regul. 3, 3–2023. 10.3389/fdsfr.2023.1323057 40980108 PMC12443087

[B7] Deghmane A. E. Taha M. K. (2022). Product review on the IMD serogroup B vaccine Bexsero(R). Hum. Vaccin Immunother. 18 (1), 2020043. 10.1080/21645515.2021.2020043 35192786 PMC8986181

[B8] Diaz-Gonzalez A. Hernandez-Guerra M. Perez-Medrano I. Sapena V. Riveiro-Barciela M. Barreira-Díaz A. (2023). Budesonide as first-line treatment in patients with autoimmune hepatitis seems inferior to standard predniso(lo)ne administration. Hepatology 77 (4), 1095–1105. 10.1097/HEP.0000000000000018 36626622

[B9] Duffy J. Marquez P. Dores G. M. Ng C. Su J. Cano M. (2020). Safety surveillance of bivalent meningococcal group B vaccine, vaccine adverse event reporting system, 2014-2018. Open Forum Infect. Dis. 7 (12), ofaa516. 10.1093/ofid/ofaa516 33324721 PMC7724509

[B10] DuVernoy T. S. Braun M. M. The V. W. G. (2000). Hypotonic–hyporesponsive episodes reported to the vaccine adverse event reporting system (VAERS), 1996–1998. Pediatrics 106 (4), e52. 10.1542/peds.106.4.e52 11015547

[B11] Evans S. J. Waller P. C. Davis S. (2001). Use of proportional reporting ratios (PRRs) for signal generation from spontaneous adverse drug reaction reports. Pharmacoepidemiol. Drug Saf. 10 (6), 483–486. 10.1002/pds.677 11828828

[B12] Griffin M. R. Braun M. M. Bart K. J. (2009). What should an ideal vaccine postlicensure safety system be? Am. J. Public Health 99 (S2), S345–S350. 10.2105/AJPH.2008.143081 19797747 PMC4504357

[B13] Hauben M. Zhou X. (2003). Quantitative methods in pharmacovigilance: focus on signal detection. Drug Saf. 26 (3), 159–186. 10.2165/00002018-200326030-00003 12580646

[B14] Herve C. Laupeze B. Del G. G. Didierlaurent A. M. Tavares D. S. F. (2019). The how's and what's of vaccine reactogenicity. NPJ Vaccines 4, 39. 10.1038/s41541-019-0132-6 31583123 PMC6760227

[B15] Howitz M. Krause T. G. Simonsen J. B. Hoffmann S. Frisch M. Nielsen N. M. (2007). Lack of association between group B meningococcal disease and autoimmune disease. Clin. Infect. Dis. 45 (10), 1327–1334. 10.1086/522190 17968829

[B16] Marshall G. S. McCormick Z. L. Johns J. S. Verduzco-Gutierrez M. Herrera-Restrepo O. Harrison L. H. (2024). Understanding the sequelae of invasive meningococcal disease in the United States. Infect. Dis. Ther. 13 (11), 2213–2220. 10.1007/s40121-024-01026-w 39269567 PMC11499580

[B17] Moro P. L. Arana J. Cano M. Lewis P. Shimabukuro T. T. (2015). Deaths reported to the vaccine adverse event reporting System, United States, 1997-2013. Clin. Infect. Dis. 61 (6), 980–987. 10.1093/cid/civ423 26021988 PMC6771280

[B18] Moro P. L. Haber P. McNeil M. M. (2019). Challenges in evaluating post-licensure vaccine safety: observations from the centers for disease control and prevention. Expert Rev. Vaccines 18 (10), 1091–1101. 10.1080/14760584.2019.1676154 31580725

[B19] O'Ryan M. Stoddard J. Toneatto D. Wassil J. Dull P. M. (2014). A multi-component meningococcal serogroup B vaccine (4CMenB): the clinical development program. Drugs 74 (1), 15–30. 10.1007/s40265-013-0155-7 24338083 PMC3890039

[B20] Olbrich K. J. Muller D. Schumacher S. Beck E. Meszaros K. Koerber F. (2018). Systematic review of invasive meningococcal disease: Sequelae and quality of life impact on patients and their caregivers. Infect. Dis. Ther. 7 (4), 421–438. 10.1007/s40121-018-0213-2 30267220 PMC6249177

[B21] Ostergaard L. Vesikari T. Absalon J. Beeslaar J. Ward B. J. Senders S. (2017). A bivalent meningococcal B vaccine in adolescents and young adults. N. Engl. J. Med. 377 (24), 2349–2362. 10.1056/NEJMoa1614474 29236639

[B22] Perez-Vilar S. Dores G. M. Marquez P. L. Ng C. S. Cano M. V. Rastogi A. (2022). Safety surveillance of meningococcal group B vaccine (Bexsero(R)), vaccine adverse event reporting system, 2015-2018. Vaccine 40 (2), 247–254. 10.1016/j.vaccine.2021.11.071 34887130 PMC9009159

[B23] Ruiz G. Y. Sohn W. Y. Seib K. L. Taha M. K. Vázquez J. A. de Lemos A. P. S. (2021). Looking beyond meningococcal B with the 4CMenB vaccine: the Neisseria effect. NPJ Vaccines 6 (1), 130. 10.1038/s41541-021-00388-3 34716336 PMC8556335

[B24] Shen S. Findlow J. Peyrani P. (2024). Global epidemiology of meningococcal disease-causing serogroups before and after the COVID-19 pandemic: a narrative review. Infect. Dis. Ther. 13 (12), 2489–2507. 10.1007/s40121-024-01063-5 39509011 PMC11582116

[B25] Shimabukuro T. T. Nguyen M. Martin D. DeStefano F. (2015). Safety monitoring in the vaccine adverse event reporting system (VAERS). Vaccine 33 (36), 4398–4405. 10.1016/j.vaccine.2015.07.035 26209838 PMC4632204

[B26] Valente P. M. O'Connor D. Galal U. Clutterbuck E. A. Robinson H. Plested E. (2020). Immunogenicity and reactogenicity of a reduced schedule of a 4-component capsular group B meningococcal vaccine: a randomized controlled trial in infants. Open Forum Infect. Dis. 7 (5), ofaa143. 10.1093/ofid/ofaa143 32494580 PMC7252280

[B27] Vigo A. Costagliola G. Ferrero E. Noce S. (2017). Hypotonic-hyporesponsive episodes after administration of hexavalent DTP-based combination vaccine: a description of 12 cases. Hum. Vaccin Immunother. 13 (6), 1–4. 10.1080/21645515.2017.1287642 28301267 PMC5489273

[B28] Zahid A. Ismail H. Wilson J. C. Grice I. D. (2025). Bioengineering outer-membrane vesicles for vaccine development: strategies, advances, and perspectives. Vaccines (Basel) 13 (7), 767. 10.3390/vaccines13070767 40733744 PMC12298926

[B29] Zorych I. Madigan D. Ryan P. Bate A. (2013). Disproportionality methods for pharmacovigilance in longitudinal observational databases. Stat. Methods Med. Res. 22 (1), 39–56. 10.1177/0962280211403602 21878461

