# Supplementary figures and images for: Post-marketing safety profile of meningococcal group B vaccines: a real-world disproportionality analysis of the VAERS database, 2015Q1–2025Q3
Source: Front Pharmacol. 2026 Mar 18;17:1745876. doi: 10.3389/fphar.2026.1745876 (PMC13038872; doi:10.3389/fphar.2026.1745876)

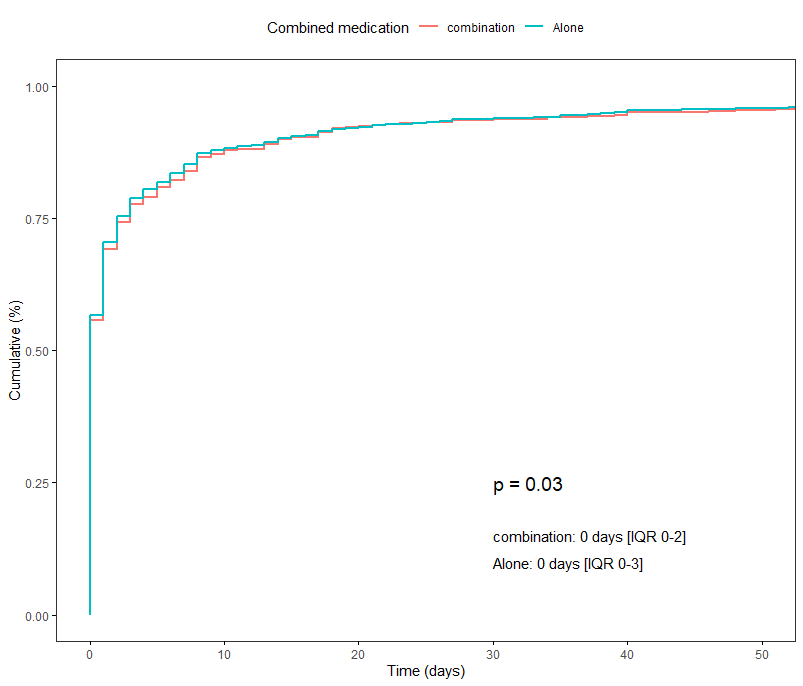

Supplement: Supplementary file 1 [file Image3.tiff]

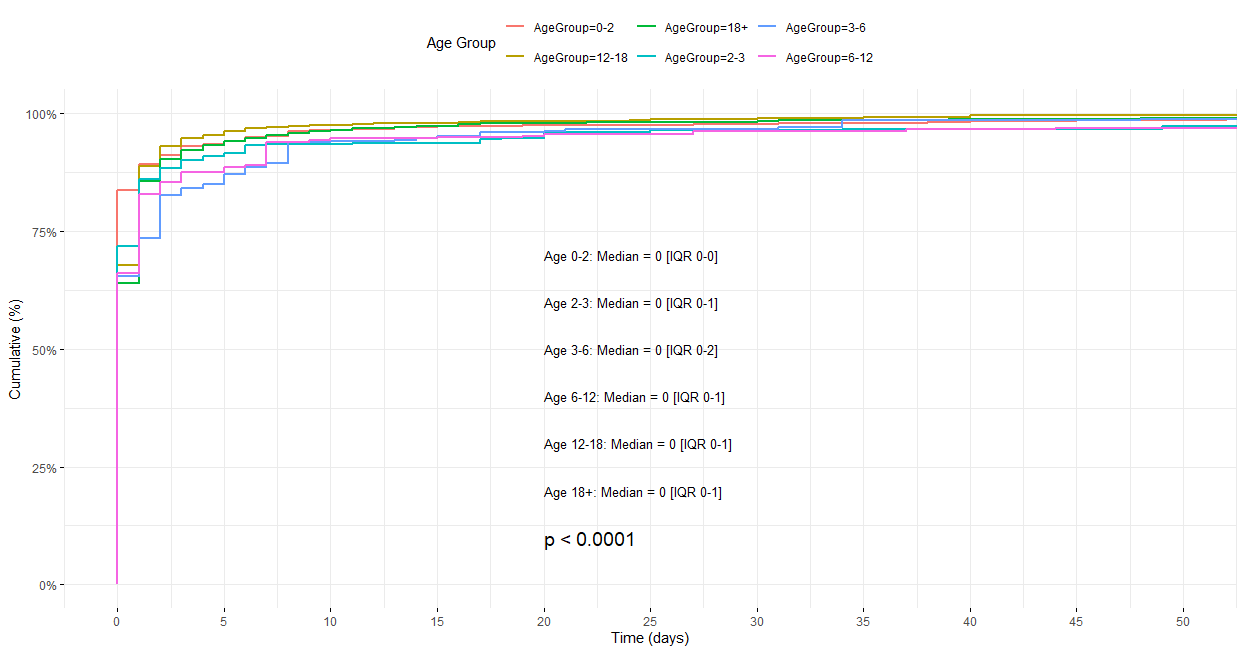

Supplement: Supplementary file 2 [file Image1.tiff]

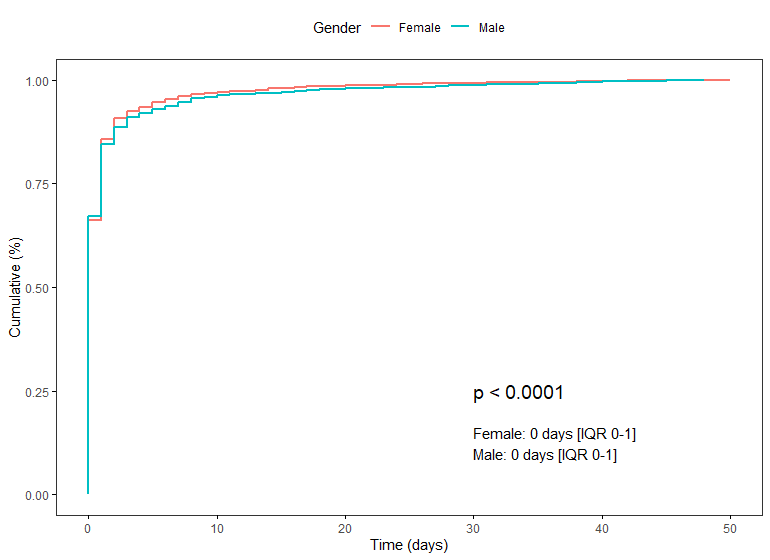

Supplement: Supplementary file 3 [file Image2.tiff]

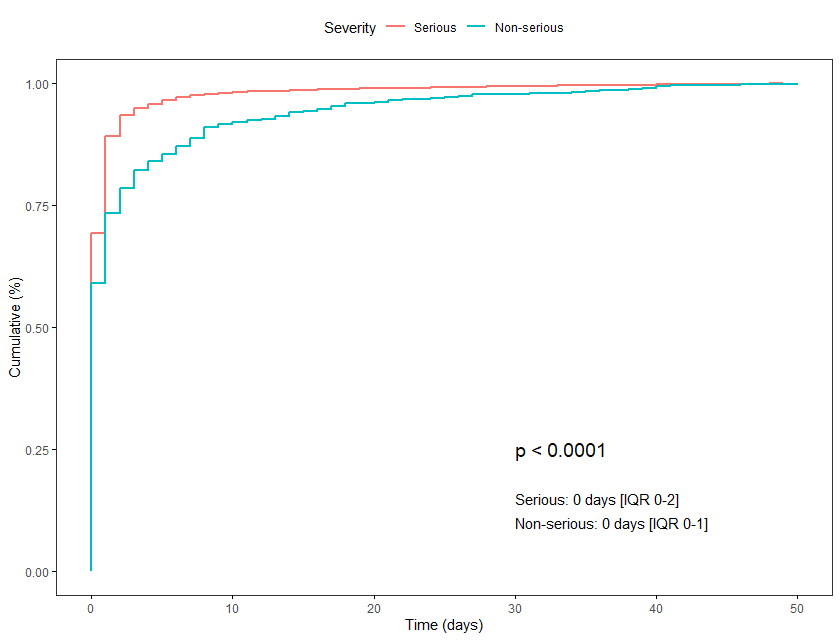

Supplement: Supplementary file 4 [file Image4.tiff]
